# Steroid-Refractory Fulminant Lymphocytic Myocarditis Successfully Treated with Tacrolimus Guided by Serial Cardiac Magnetic Resonance Imaging

**DOI:** 10.1016/j.cjco.2026.05.003

**Published:** 2026-05-14

**Authors:** Yu Agata, Atsushi Tada, Satonori Tsuneta, Noriyuki Otsuka, Utano Tomaru, Rui Katano, Kenta Otsuka, Yutaro Yasui, Toshiyuki Nagai, Kohsuke Kudo, Shinya Tanaka, Toshihisa Anzai

**Affiliations:** a Department of Cardiovascular Medicine, Faculty of Medicine and Graduate School of Medicine, Hokkaido University, Sapporo, Japan; b Department of Radiology, Graduate School of Dental Medicine, Hokkaido University, Sapporo, Japan; c Department of Surgical Pathology, Hokkaido University Hospital, Sapporo, Japan; d Department of Cancer Pathology, Faculty and Graduate School of Medicine, Hokkaido University, Sapporo, Japan

**Keywords:** fulminant myocarditis, lymphocytic myocarditis, tacrolimus, cardiac magnetic resonance, steroid-refractory

**We report a case of fulminant nonviral lymphocytic myocarditis in a 37-year-old woman. Although she responded to an initial steroid pulse therapy, relapse occurred under high-dose maintenance prednisolone and proved refractory to a second steroid pulse. The addition of tacrolimus successfully controlled the recurrent inflammation. Serial cardiac magnetic resonance imaging was used to assess disease activity and guide safe tapering of immunosuppression. This case highlights the potential role of calcineurin inhibitors in steroid-refractory fulminant myocarditis and the value of serial cardiac magnetic resonance imaging as a noninvasive decision-making tool for longitudinal management of refractory myocarditis.**

A 37-year-old woman with no past medical history presented to a referral hospital with sudden chest oppression and dyspnea, without symptoms of a preceding infection. An electrocardiogram (ECG) showed ST-segment elevation in the inferior leads, with an elevated troponin level. Transthoracic echocardiography (TTE) revealed pericardial effusion without regional wall-motion abnormalities, and acute pericarditis was suspected after coronary computed tomography excluded ischemic heart disease. Three days later, her dyspnea worsened, with follow-up TTE showing a marked decline in left ventricular ejection fraction (LVEF) to 35%, with progression of pericardial effusions. She became hypotensive and hypoxic, requiring intravenous dobutamine infusion, and she was transferred to our university hospital after intra-aortic balloon pump insertion for suspected acute myocarditis with hemodynamic instability.

Upon arrival, her blood pressure was 100/59 mm Hg, her heart rate was 122 beats per minute, and her oxygen saturation was 98% on room air. Laboratory testing revealed markedly elevated levels of cardiac biomarkers, including troponin T (1863 ng/L) and NT-proBNP (6396 pg/mL). Peripheral blood analysis showed a normal eosinophil count (2.4%), and she had no history of drug allergies. Chest radiography showed cardiomegaly, pulmonary congestion, and right pleural effusion (Fig. 1A). ECG revealed low voltage in the limb leads and poor R-wave progression in the precordial leads (Fig. 1B). TTE demonstrated diffuse biventricular hypokinesis with a severely reduced LVEF of 29% (Fig. 1, C and D; Video 1A, view video online).

**Figure 1:**
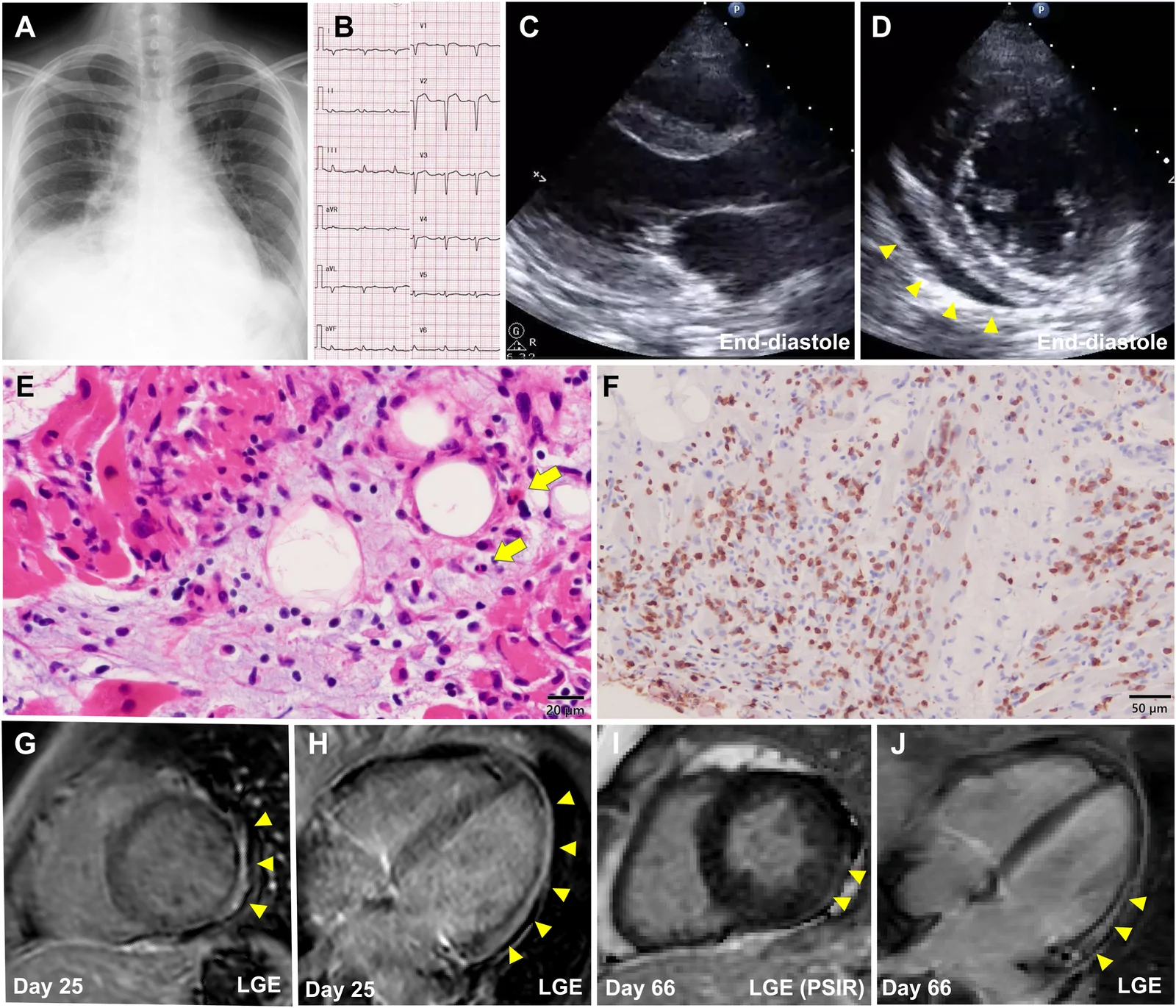
(**A**) Chest radiography showing right-dominant pleural effusion, pulmonary congestion, and mild cardiomegaly. (**B**) Electrocardiogram demonstrating poor R-wave progression in precordial leads V1-3 and negative T-waves in inferior leads II, III, and aVF. (**C, D**) Transthoracic echocardiography at end-diastole revealing diffuse biventricular hypokinesis with a left ventricular ejection fraction of 29%. Pericardial effusion is also observed (**arrowheads**). (**E**) Hematoxylin and eosin staining of the right ventricular endomyocardial biopsy specimen reveals myocardial degeneration and fragmentation with extensive interstitial infiltration of inflammatory cells. The infiltrate consists of a mixture of lymphocytes, histiocytes, and eosinophils (**arrows**), accompanied by prominent edema and myocytolysis. (**F**) CD3 immunostaining from the final pathology report (issued on day 8) demonstrating that the infiltrating inflammatory cells are predominantly T-lymphocytes. (**G, H**) Late gadolinium enhancement (LGE) cardiac magnetic resonance images obtained on day 25 showing band-like subepicardial enhancement involving the lateral wall from the base to the apex (**arrowheads**). **(I, J)** Follow-up LGE cardiac magnetic resonance images obtained on day 66. The short-axis view (**I**) was acquired using a phase-sensitive inversion recovery (PSIR) sequence to minimize motion artifacts. Both images demonstrate a clear regression of LGE, compared to the findings on day 25.

Despite dobutamine therapy and intra-aortic balloon pump support, emergency right heart catheterization demonstrated severe cardiogenic shock, with a cardiac index of 1.38 L/min/m^2^, mean pulmonary capillary wedge pressure of 24 mm Hg, and mixed venous oxygen saturation of 44.3%. Progressive agitation due to hypoperfusion required intubation and the simultaneous initiation of veno-arterial extracorporeal membrane oxygenation and Impella CP (Abiomed, Danvers, MA) support to provide both systemic circulatory support and left ventricular unloading.

Rapid histologic examination revealed cardiomyocyte necrosis with interstitial inflammatory infiltrates composed predominantly of lymphocytes and histiocytes, along with scattered eosinophils (Fig. 1E). Although eosinophil infiltration was sparse, eosinophilic myocarditis could not be excluded, given the presence of these cells and the absence of a viral prodrome. Considering the life-threatening presentation and the high likelihood of steroid responsiveness in such cases, intravenous methylprednisolone pulse therapy (1000 mg/d for 3 consecutive days) was initiated promptly (Fig. 2). The clinical response was favourable, with rapid hemodynamic stabilization allowing discontinuation of veno-arterial extracorporeal membrane oxygenation on day 4, and Impella CP support on day 6. Cardiac biomarkers declined promptly, ECG showed recovery of R-wave amplitude, and LVEF improved to 59% (Video 1B, view video online). Maintenance oral prednisolone (PSL) was started at 50 mg/d (1.0 mg/kg/d), tapering by 5 mg per week.

**Figure 2:**
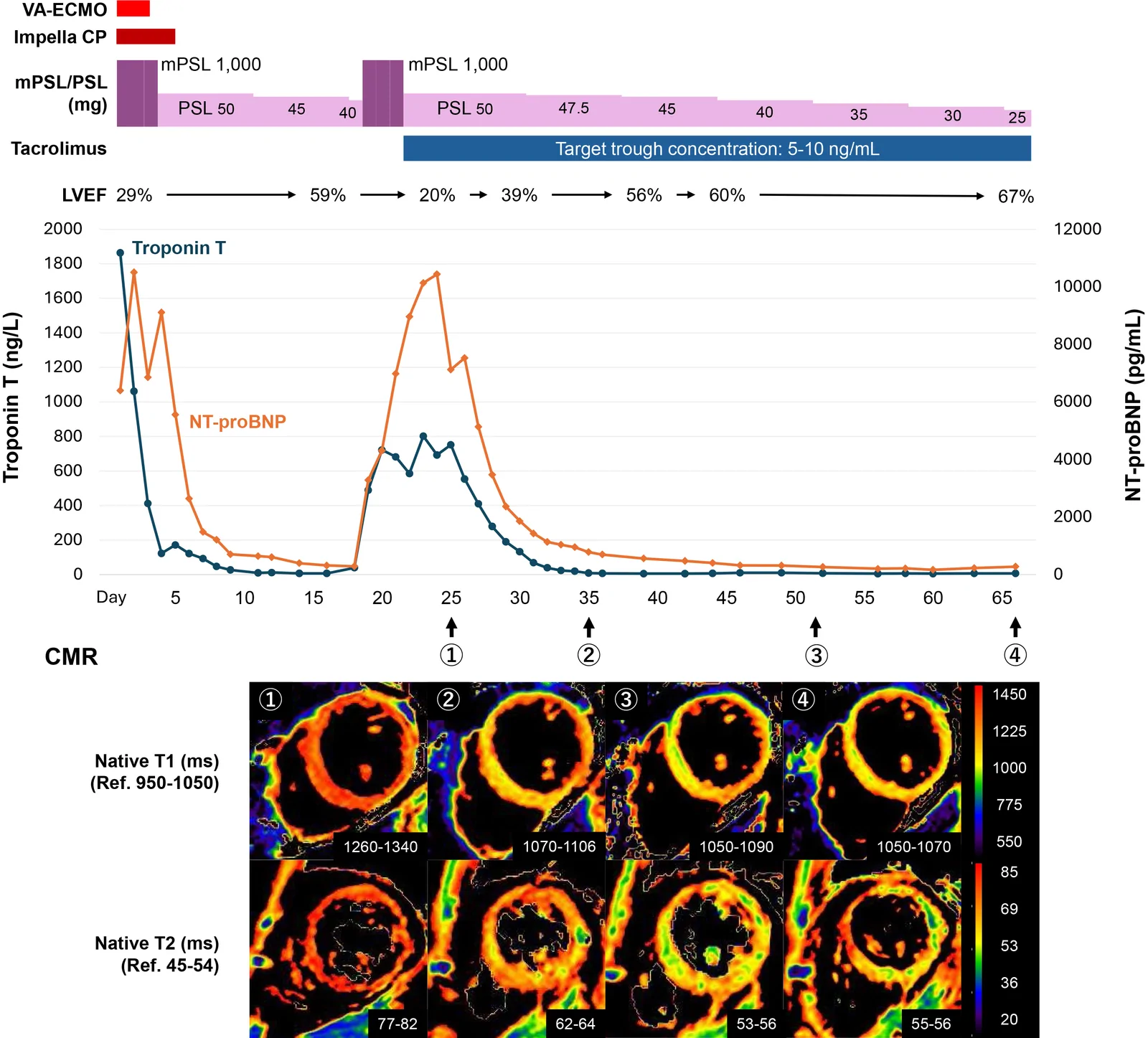
Clinical course and serial cardiac magnetic resonance (CMR) findings. The **upper timeline** depicts the duration of mechanical circulatory support and the administration of methylprednisolone (mPSL), prednisolone (PSL), and tacrolimus. The **middle panel** shows the serial changes in serum troponin T (**navy line**) and N-terminal pro-brain natriuretic peptide (NT-proBNP; **orange line**), and left ventricular ejection fraction (LVEF). The **lower panel** displays serial native T1 and T2 mapping images obtained at 4 time points (day 25, 35, 52, and 66). Both parameters demonstrate a progressive visual and quantitative reduction in values, reflecting the resolution of myocardial inflammation. Impella CP is from Abiomed, Danvers, MA. Ref., reference; VA-ECMO, veno-arterial extracorporeal membrane oxygenation.

On day 19, while receiving PSL 40 mg/d, she developed fever, chest tightness, and dyspnea, with ECG showing recurrent low voltage and new ST-segment depression in the precordial lead, and her troponin T level had increased to 422 ng/L (Fig. 2). Right heart catheterization showed preserved hemodynamics, with a cardiac index of 2.78 L/min/m^2^, mean pulmonary capillary wedge pressure of 10 mm Hg, and mixed venous oxygen saturation of 63.7%, obviating the need for mechanical circulatory support. Nonetheless, persistent elevation of biomarkers with worsening left ventricular dysfunction indicated a steroid-refractory relapse of myocarditis.

A second course of pulse therapy proved ineffective, with plateauing troponin and N-terminal pro-brain natriuretic peptide levels and progressive decline in LVEF (Fig. 2; Video 1C, view video online). The final pathologic report demonstrated sparse eosinophilic infiltration (≈4/high-power field in hot spots) with predominant lymphocytic inflammation, consistent with lymphocytic myocarditis (Fig. 1F), and viral polymerase chain reaction testing was negative. A point to note is that a low fraction of eosinophils may be observed as a nonspecific finding in ongoing severe inflammation—especially when interventions or treatments precede the biopsy—and does not contradict a predominant lymphocytic process. A hematological workup, including bone marrow aspiration and chromosomal analysis, showed no evidence of eosinophilic leukemia or fusion genes associated with hypereosinophilic syndrome. Systemic autoimmune screening, including antinuclear antibody and rheumatoid factor, and antineutrophil cytoplasmic antibody (ANCA), was negative. In addition, consultation with rheumatology specialists revealed no clinical evidence of systemic autoimmune disease, including eosinophilic granulomatosis with polyangiitis. Based on these findings and the steroid-refractory disease course, fulminant nonviral lymphocytic myocarditis with relapse was diagnosed. Accordingly, tacrolimus (targeting a trough concentration of 5-10 ng/mL), a calcineurin inhibitor (CNI), was added on day 22 as rescue immunosuppressive therapy (Fig. 2), in accordance with the Japanese Circulation Society guidelines.^1^ Tacrolimus was initiated via continuous intravenous infusion for 2 days and subsequently was transitioned to oral administration with the dose titrated between 2.5 and 4.7 mg/d. Trough concentrations were maintained between 4.6 and 9.5 ng/mL. Mild finger tremor was observed but did not require dose modification or discontinuation. Following tacrolimus initiation, cardiac biomarker levels markedly declined, and LVEF showed a gradual and sustained recovery from 20% (Fig. 2; Video 1D, view video online).

Cardiac magnetic resonance (CMR) imaging on day 25 using a 1.5-Tesla scanner demonstrated globally prolonged native T1 and T2 values (obtained via modified Look-Locker inversion recovery [MOLLI] and gradient and spin-echo [GRASE] sequences, respectively; Fig. 2), along with band-like subepicardial late gadolinium enhancement (LGE) involving the lateral wall (Fig. 1, G and H), consistent with active myocardial inflammation. Thereafter, serial CMR examinations were used as surrogate markers of myocardial inflammatory activity to guide immunosuppressive tapering, alongside cardiac biomarkers and echocardiography (Supplemental Table S1). Follow-up CMR studies on days 35, 52, and 66 showed progressive normalization of T1/T2 values and partial regression of LGE with residual enhancement observed at discharge (Fig. 1, I and J and Fig. 2). The slight variation in T2 values at day 66 was regarded as measurement variability rather than inflammation, given the stable clinical, biomarker, and echocardiographic findings.

The patient was discharged on day 67 on PSL 25 mg/d. Outpatient tapering proceeded uneventfully under close monitoring of clinical symptoms, biomarkers, and echocardiography. PSL was discontinued successfully on day 170, without any signs of recurrence. The patient remains in stable clinical condition with a functional status of New York Heart Association class I.

## Discussion

We present a case of fulminant nonviral lymphocytic myocarditis that initially responded to corticosteroid pulse therapy but relapsed under high-dose maintenance corticosteroids. The recurrent inflammation was refractory to a second course of pulse therapy but was successfully controlled by the addition of tacrolimus. This case highlights the potential efficacy of concurrent CNI therapy for relapsing or refractory myocarditis, as well as the critical utility of serial CMR monitoring.

Although supportive care remains the mainstay for lymphocytic myocarditis, immunosuppressive therapy, including corticosteroid pulse therapy, is considered specifically for fulminant, nonviral cases. Although the **T**ailored **Im**munosuppression in Virus-Negative **I**nflammatory **C**ardiomyopathy (TIMIC) trial demonstrated the efficacy of immunosuppressive therapy in chronic virus-negative lymphocytic myocarditis,^2^ evidence supporting its use in fulminant and relapsing disease remains limited. Although Hiruma et al. reported CNI success for relapses during PSL tapering at 10-15 mg/d,^3^ our patient relapsed at a much higher dose (0.8 mg/kg/d). In such high-risk scenarios, serial CMR provided the objective evidence necessary for safe tapering of immunosuppression. Although recent European Society of Cardiology guidelines recommend azathioprine, mycophenolate mofetil, or cyclosporine for refractory cases,^4^ the Japanese Circulation Society guidelines specifically include tacrolimus, based on accumulated clinical experience in Japan.^1^ We selected tacrolimus, another CNI, to avoid cyclosporine-associated adverse events.

Although recurrence of fulminant lymphocytic myocarditis has been documented, previous relapses typically occurred after corticosteroid withdrawal or during low-dose maintenance therapy. To our knowledge, relapses under high-dose prednisolone (40 mg/d) have not been reported previously, suggesting a highly active and potentially steroid-resistant underlying autoimmune mechanism in this patient. Given the marked T-lymphocyte infiltration observed on biopsy, the CNI likely contributed to disease control by suppressing the transcription of proinflammatory cytokines.

Assessing ongoing inflammatory activity is challenging in refractory myocarditis. Because repeated endomyocardial biopsies are invasive, serial CMR evaluation played a central role in the therapeutic management of this patient. Native T1 and T2 mapping, along with the regression of LGE—likely reflecting the resolution of acute interstitial edema and transient myocyte injury—sensitively reflected myocardial inflammatory activity.^5^ Their normalization, interpreted as a surrogate for histologic resolution, guided safe tapering of PSL while minimizing relapse risk. Notably, the residual LGE at discharge most likely represents replacement fibrosis rather than ongoing active inflammation. Although the absence of cardiac autoantibody testing, human leukocyte antigen typing, and complement assessment is a limitation, this case highlighted the importance of CMR as a practical decision-making tool for use in the longitudinal management of refractory myocarditis.

**Novel Teaching Points**

• Tacrolimus may serve as an effective rescue therapy for fulminant, viral-negative lymphocytic myocarditis refractory to high-dose corticosteroid therapy.
• Serial CMR with T1 and T2 mapping provides a noninvasive tool to assess inflammatory activity and guide the safe tapering of immunosuppression without repeated biopsies.
• Myocarditis relapse during high-dose PSL therapy may indicate highly aggressive, steroid-resistant disease requiring early intensification of treatment.

## Ethics Statement

The research reported in this paper adhered to CARE guidelines.

## Patient Consent

The authors confirm that patient consent forms have been obtained for this article.

## Funding Sources

The authors have no funding sources to declare.

## Disclosures

The authors have no conflicts of interest to disclose.

## Supplementary Material

Supplementary PDF

Video 1 Serial transthoracic echocardiography findings. The upper panels display the parasternal long-axis views, and the lower panels display the parasternal short-axis views at the mid-ventricular level. **(A)** On admission (Day 1): Severe left ventricular dysfunction with a left ventricular ejection fraction (LVEF) of 29%. **(B)** Before relapse (Day 16): Recovered left ventricular function with an LVEF of 59%. **(C)** After relapse (Day 24): Recurrent severe left ventricular dysfunction with an LVEF of 20%. **(D)** Before discharge (Day 66): Normalized left ventricular function with an LVEF of 67%. LVEF, Left ventricular ejection fraction.
