# Supplementary material for: Steroid-Refractory Fulminant Lymphocytic Myocarditis Successfully Treated with Tacrolimus Guided by Serial Cardiac Magnetic Resonance Imaging
Source: CJC Open. 2026 May 14;8(9):1183–7. doi: 10.1016/j.cjco.2026.05.003 (PMC13583580; doi:10.1016/j.cjco.2026.05.003)
Supplement: Supplementary file 1 [file mmc1.pdf]

**Supplemental Table S1**: Timeline of immunosuppressive therapy, cardiac biomarkers, and cardiac magnetic resonance findings.

| *Day* | *PSL (mg/day)* | *mPSL (mg/day)* | *Tacrolimus dose (mg/day)* | *Tacrolimus trough (ng/mL)* | *LVEF (%)* | *Troponin T (ng/L)* | *NT-proBNP (pg/mL)* | *Native T1 (ms)* | *Native T2 (ms)* | *LGE extent [1] (Visual Estimate)* |
|---|---|---|---|---|---|---|---|---|---|---|
| *1* | – | 1,000 | – | – | 29 | 1,863 | 6,396 | – | – | – |
| *2* | – | 1,000 | – | – | – | 1,062 | 10,503 | – | – | – |
| *3* | – | 1,000 | – | – | – | 412 | 6,855 | – | – | – |
| *4* | 50 | – | – | – | – | 123 | 9,109 | – | – | – |
| *11* | 45 | – | – | – | – | 10 | 646 | – | – | – |
| *16* | 45 | – | – | – | 59 | 7 | 318 | – | – | – |
| *18* | 40 | – | – | – | – | 40 | 288 | – | – | – |
| *19* | – | 1,000 | – | – | – | 489 | 3286 | – | – | – |
| *20* | – | 1,000 | – | – | – | 722 | 4311 | – | – | – |
| *21* | – | 1,000 | – | – | – | 682 | 6,978 | – | – | – |
| *22* | 50 | – | 0.5 (IV) | – | – | 585 | 8,962 | – | – | – |
| *23* | 50 | – | 0.6 (IV) | 3.1 | – | 802 | 10,133 | – | – | – |
| *24* | 50 | – | 2.5 (PO) | 3.5 | 20 | 693 | 10,436 | – | – | – |
| *25* | 50 | – | 2.7 (PO) | 5 | – | 752 | 7,125 | 1260-1340 | 77-82 | Marked (Baseline, 100%) |
| *30* | 47.5 | – | 3.7 (PO) | 6.5 | 39 | 133 | 1,863 | – | – | – |
| *31* | 47.5 | – | 3.2 (PO) | – | – | 69 | 1,430 | – | – | – |
| *35* | 47.5 | – | 4.0 (PO) | 4.6 | – | 9 | 784 | 1070-1106 | 62-64 | Moderate (~70%) |
| *38* | 45 | – | 4.5 (PO) | 5.4 | – | – | – | – | – | – |
| *39* | 45 | – | 4.5 (PO) | 6.6 | 56 | 6 | 562 | – | – | – |
| *45* | 40 | – | 4.7 (PO) | – | 60 | – | – | – | – | – |
| *52* | 35 | – | 4.7 (PO) | 7.4 | – | 8 | 266 | 1050-1090 | 53-56 | Mild (~50%) |
| *59* | 30 | – | 4.4 (PO) | – | – | – | – | – | – | – |
| *66* | 25 | – | 4.4 (PO) | 6.8 | 67 | 7 | 277 | 1050-1070 | 55-56 | Mild (~50%) |

1) Percentages for late gadolinium enhancement (LGE) extent represent subjective visual estimates relative to the maximal inflammatory burden observed on Day 25 (set as 100%).

IV, intravenous; LGE, Late gadolinium enhancement; LVEF, Left ventricular ejection fraction; mPSL, Methylprednisolone; NT-proBNP, N-terminal pro-brain natriuretic peptide; PO, per os; PSL, Prednisolone.
